# Comparison of morphology, development and expression patterns of *hsf* and *hsp11.0* of *Cotesia chilonis* under normal and high temperature

**DOI:** 10.7717/peerj.11353

**Published:** 2021-04-27

**Authors:** Fu-Jing He, Feng Zhu, Ming-Xing Lu, Yu-Zhou Du

**Affiliations:** 1 School of Horticulture and Plant Protection & Institute of Applied Entomology, Yangzhou University, Yangzhou, P. R. China; 2 Plant Protection and Quarantine Station of Jiangsu Province, Nanjing, P. R. China; 3 Joint International Research Laboratory of Agriculture and Agri-Product Safety, Yangzhou University, Yangzhou, P. R. China

**Keywords:** *Cotesia chilonis*, high temperature, growth, reproduction, developmental stages

## Abstract

*Cotesia chilonis* (Munakata) is the dominant parasitic wasp of the rice pest, *Chilo suppressalis* (Walker), and is a valuable parasitic wasp for the prevention and control of *C. suppressalis*. In this study, developmental indicators and expression of *Cchsp11.0* (heat shock protein 11.0) and *Cchsf* (heat shock factor) were compared for *C. chilonis* at 27 °C and 36 °C. Developmental duration, morphology, emergence rate, and number of *C. chilonis* offspring were shortened at 36 °C while the ratio of females to males increased. *Cchsp11.0* and *Cchsf* were highly expressed in the 1^st^ instar stage at 36 °C, and *Cchsp11.0* expression gradually decreased as *C. chilonis* matured; *Cchsf* expression was not correlated with *Cchsp11.0* expression. Compared with 27 °C, the expression pattern of *Cchsp11.0* and *Cchsf* was also not consistent, and *Cchsp11.0* expression increased significantly at the adult stage. In conclusion, mildly high temperatures impact growth, development and reproduction of *C. chilonis* and stimulate the expression of *Cchsp11.0* and *Cchsf*, and *Cchsp11.0* and *Cchsf* play different roles in different developmental stages of *C. chilonis* at normal and high temperature.

## Introduction

*Chilo suppressalis* (Walker) (Lepidoptera: Pyralidae) is a detrimental rice pest in southern Europe, northern Africa and widely distributed in China and other Asian countries (Lu et al., 2013; Luo et al., 2014). Issues of pesticide residues, environmental pollution and pesticide poisoning have attracted the attention and stimulated the explore on new biological control methodologies such as the use of natural enemies (He et al., 2013; Hong, 2015; Gao et al., 2019). *Cotesia chilonis* (Munakata) is the dominant parasitoid of *C. suppressalis* and occurs in southeastern and eastern Asia, regions where it has value as a biological control agent for *C. suppressalis* (Huang, Wu & Ye, 2011; Wu et al., 2013; Pan et al., 2018*)*. It is notable that the parasitism rate for *C. chilonis* on overwintering *C. suppressalis* larvae can be as high as 90% (Hang, Chen & Wu, 1989; Pan, 2018).

With the onset of global warming, climatic extremes have garnered widespread attention (Christidis & Stott, 2015; Palmer, 2014). Extreme shifts in temperature can induce molecular, biochemical, and physiological changes that alter organismal fitness, insect phenology and temperature-dependent population dynamics (Ma & Ma, 2020). However, most studies of insect responses to climate extremes have focused on herbivorous pests, less attention has been paid to the response of natural enemies such as parasitoids and predators. Previous research has indicated that many parasitoids may be more sensitive to heat than herbivorous pests and plant hosts due to direct and indirect effects of high temperatures (Agosta, Joshi & Kester, 2018; Van Baaren, Le Lann & Van Alphen, 2010). In addition, previous studies have also shown that temperature extremes may impact parasitoid survival, development, and reproduction (Chen et al., 2019; Flores-Mejia et al., 2016). For example, exposure to extreme temperatures commonly results in higher egg-to-larval mortality and reduced larval growth (Rocha et al., 2017; Zhou et al., 2018; Potter, Davidowitz & Arthur Woods, 2011).

Insects generally exhibit behavioral and physiological responses to high temperatures to mitigate adverse effects (González-Tokman et al., 2020). For example, insects produce heat shock proteins (HSPs) to prevent protein denaturation and to protect themselves when exposed to high temperature (Kim, Kim & Kim, 1998; Aeverman & Waters, 2008; Mayra & Silvina, 2018). The regulation of HSPs in response to temperature stress is regulated by heat shock transcription factors (HSFs) (Akerfelt, Morimoto & Sistonen, 2010).

Therefore, in this study, we asked: ‘What impacts do high summer temperatures have on *C. chilonis* in the field?’ To investigate, we chose a moderately high temperature (36 °C) based on previous research (Pan et al., 2018). This temperature was used to simulate field temperatures that *C. chilonis* may encounter in nature and to explore the effect of high temperature stress on growth, development, and reproduction. Moreover, the expression of *Cchsp11.0* and *Cchsf* in *C. chilonis* was studied in order to indicate the regulation of HSP and HSF of *C. chilonis* responded to global warming.

## Materials and Methods

### Insects

*C. suppressalis* and *C. chilonis* were collected from a suburb of Yangzhou (32.39°N, 119.42°E) and reared in the laboratory at 27 ± 1 °C, 60–70% RH and a 16:8 h (light/dark) photoperiod (Pan et al., 2018). *C. suppressalis* larvae were supplied with an artificial diet (Gao et al., 2019). *C. chilonis* adults were fed with a 10% honey/water solution and propagated using 5^th^ instar larvae of *C. suppressalis* as hosts.

### Sample treatments

A single 5^th^ instar of *C. suppressalis* was placed in a test tube and two female and one male *C. chilonis* adults were added for breeding by syngamy while only two unfertilized female *C. chilonis* adults were added for breeding by parthenogenesis. Insects were incubated for 6 h at 27 °C in darkness to facilitate parasitism of *C. suppressalis* with 10% honey for breeding, and a single 5^th^ instar of *C. suppressalis* is parasitized only once; once parasitism occurred, *C. suppressalis* larvae were allowed to feed on an artificial diet. A subset of the parasitized *C. suppressalis* was incubated at 36 °C from 10:00 am to 2:00 pm daily to simulate the high temperatures encountered in the field; insects were maintained at 27 °C the remaining hours of the day.

Insects were maintained using the two different temperature regimes described above until *C. chilonis* emerged from *C. suppressalis*. *C. chilonis* adults are reared with 10% honey. Three parasitized *C. suppressalis* were dissected from the two treatments on a daily basis, and the development of five randomly-selected *C. chilonis* individuals was inspected; this included photographing and recording body length, head width and instar stage. Photos were taken with a KEYENCE VHX-5000 system and optimized by Adobe Photoshop CS6. In addition, different developmental stages of *C. chilonis* (egg, 1^st^ instar, 2^nd^ instar, 3^rd^ instar, pupa, adult) were collected and stored at −80 °C until needed. Treatments either contained 30 (egg and instars) or five individuals (adult and pupa). All treatments were replicated three times.

### RNA extraction

Total RNA was extracted from *C. chilonis* using the RNA-easy^™^ Isolation Reagent (Vazyme, Nanjing China). The integrity of RNA was verified by comparing ribosomal RNA bands in ethidium bromide-stained gels, and RNA purity was examined using spectrophotometric measurements at A_260_ and A_280_ nm (NanoDrop One, Thermo Fisher Scientific, Waltham, MA, USA).

### Real-time qPCR

Total RNA was isolated from the different treatments as described above, and the Bio-Rad iScript^™^ cDNA Synthesis Kit (Bio-Rad, Irvine, CA, USA) was used to reverse transcribe 0.5 µg total RNA into first strand cDNA. The primers used for real-time quantitative PCR (Table 1) were designed based on the full-length cDNA sequence of genes. Real-time PCR reactions were conducted using SYBR Green I in a 20 μl volume that included 10 μl iTaq^™^ SYBR^®^ Green Supermix, 6 μl ddH_2_0, 2 μl cDNA template and 1 μl each of the corresponding forward and reverse primers. Reaction conditions for PCR were as follows: 3 min initial denaturation step at 95 °C, followed by 40 cycles of 15 s denaturation at 95 °C, and 30 s annealing at the Tm for each gene (Table 1). Melting curve analysis was carried out to evaluate the homogeneity of the amplified PCR products. Each PCR reaction was replicated in triplicate. *H3* was regarded as the reference gene (Li et al., 2019).

**Table 1:** Primers used for qRT-PCR analysis.

Gene	Primer sequences (5′→3′)	Tm (°C)	Length (bp)
*hsp11.0*	F: ACAAAGTTCTCCTCCCCG	59.4	90
	R: GCAACAATGTCTGATTCACG		
*hsf*	F: TTAGGTGCTGAAAGTGCCGA	60.0	191
	R: AGTACGCAAGTCGAGCTGAA		
*H3*	F: CGTCGCTCTTCGTGAAATCA	58.1	122
	R: TCTGGAAACGCAAGTCGGTC		

### Statistical analysis

Relative quantitative analysis was performed by the 2^−ΔΔCt^ method to obtain expression levels. Differences in mean values were analyzed using one-way ANOVA and the independent-sample *t*-test. Homogeneity of variances among treatments was measured by Levene’s test, and significance was assessed by Tukey’s test. All statistics were performed using SPSS 16.0 software and shown as means ± SE (standard error).

## Results

### Different developmental stages of *C. chilonis*

*C. chilonis* matured through four developmental phases including egg, larval, pupal and adult stages, and according to their body length and head width, the larvae are divided into three larval instars, L1, L2 and L3 (O’Donnell, 1987; Shaw & Huddleston, 1991; Carignan, Boivin & Stewart, 1995; Li, 2011). At 27 °C, the duration of each phase was approximately 3 days for eggs, 2–3 days for L1, 1-2 days for both L2 and L3, 4-5 days for pupae and 3–4 days for adults.

**Figure 1:**
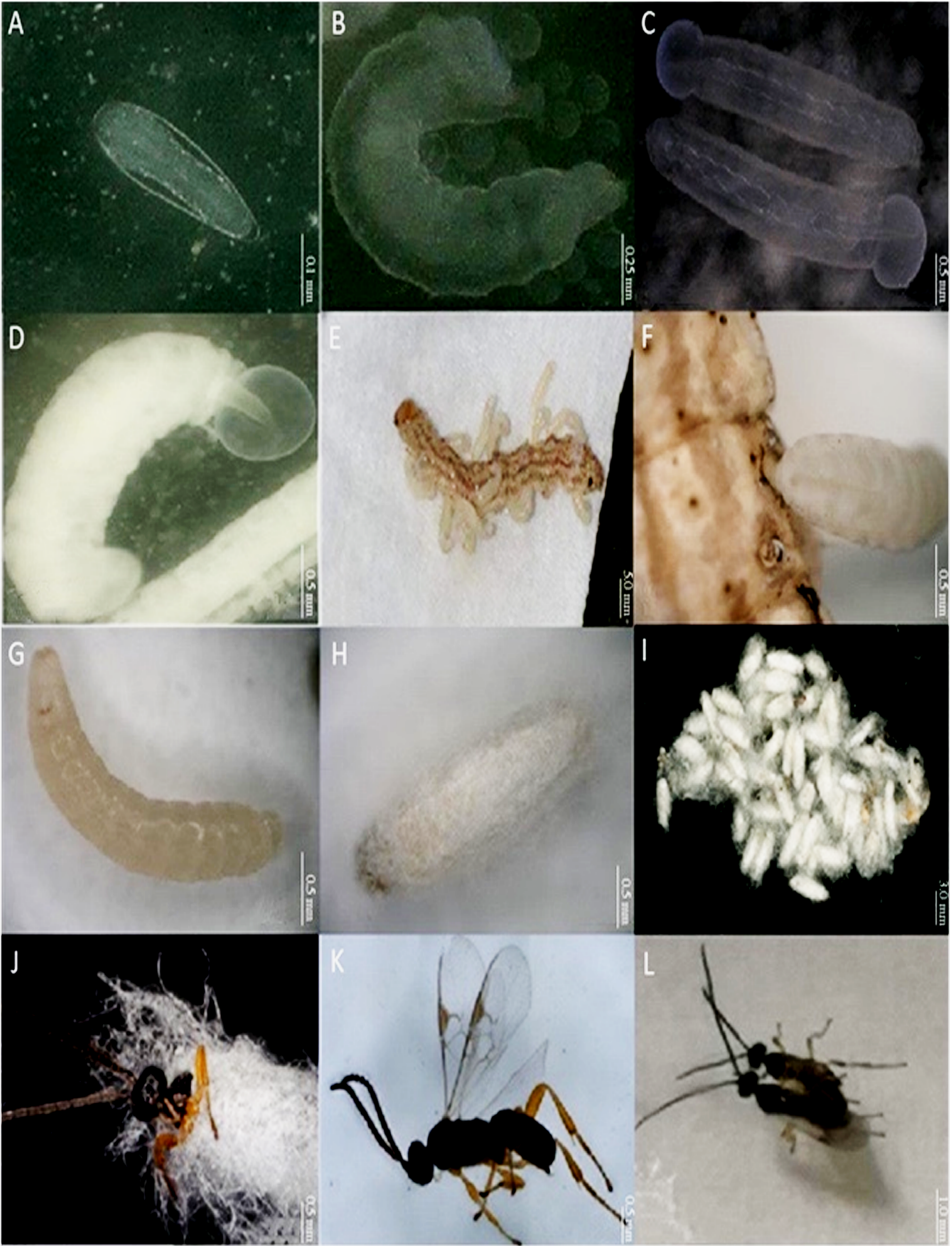
Different developmental stages of *Cotesia chilonis* at 27 °C. (A) Egg. (B) First instar larvae. (C) Second instar larvae. (D) Third instar larvae. (E) and (F) show emergence of 3^rd^ instar *C. chilonis* larvae from the host, *C. suppressalis*. (G) Inner pupa. (H) and (I) Cocoons. (J) Emergence of *C. chilonis* adults from cocoons. (K) Adult female. (L) Copulation of female (right) and male (left) adults.

For egg stage, developmental time from initial parasitism to completion of the egg stage was 72 h; at this time point, eggs were located in the hemolymph of *C. suppressalis* larvae (Fig. 1A). The length of eggs was 0.37 ± 0.01 mm; the shape was hymenopteriform with elongate-oval, transparent heads that were larger and broader than thoraces and abdomens. Giant cells began to appear on the 2^nd^ day after parasitism by syngamy or on the 3^rd^ day after parasitism by parthenogenesis.

For larval stage, the larval stage of *C. chilonis* can be divided into 1^st^, 2^nd^, and 3^rd^ larval instar stage (Figs. 1B–1D). All three instars can co-exist in fifth instar larvae of *C. suppressalis*; however, only the 3^rd^ instar emerges from the host for pupation (Li, 2011). The duration of the 1^st^, 2^nd^ and 3^rd^ larval instar stages were 3.16 ± 0.21 days, 2.70 ± 0.20 days and 1.55 ± 0.12 days at 27 °C, respectively. Body lengths of 1^st^, 2^nd^ and 3^rd^ instar *C. chilonis* larvae were 1.30 ± 0.08 mm, 2.63 ± 0.04 mm and 3.23 ± 0.08 mm, and head widths were 0.31 ± 0.02 mm, 0.56 ± 0.01 mm and 0.57 ± 0.02 mm, respectively (Table 2).

**Table 2:** Developmental indicators of different developmental stages of *Cotesia chilonis*.

	Developmental duration (days)		Body length (mm)		Head width (mm)	
	27 °C	36 °C	27 °C	36 °C	27 °C	36 °C
Eggs	2.00 ± 0.00	2.00 ± 0.00	0.37 ± 0.01	0.45 ± 0.01	/	/
1^st^ larvae	3.16 ± 0.20	4.07 ± 0.21	1.30 ± 0.08	1.29 ± 0.06	0.31 ± 0.02	0.30 ± 0.02
2^nd^ larvae	2.70 ± 0.20	2.35 ± 0.21	2.63 ± 0.04	2.62 ± 0.01	0.56 ± 0.01	0.54 ± 0.01
3^rd^ larvae	1.55 ± 0.12	0.12 ± 0.14	3.23 ± 0.08	3.09 ± 0.04	0.57 ± 0.02	0.54 ± 0.02
Pupae	1.87 ± 0.35	1.93 ± 0.09	/	/	/	/
Adults	5.28 ± 0.07	4.46 ± 0.09	/	/	/	/

For pupal stage, third instar larvae of *C. chilonis* gnawed their way out of *C. suppressalis* (Fig. 1E). The surface of *C. chilonis* was glossy and milky upon emergence (Fig. 1F) and then developed into a beige-colored pupa within 24 h that was ultimately encased in a cocoon (Figs. 1G, 1H). Cocoons were white and approximately 2–3 mm long and 1 mm in diameter; they were often clustered in groups of 20–50 (Fig. 1I). The number of cocoons produced by a single parasitic *C. suppressalis* was 42.00 ± 6.87 at 27 °C. Clusters of cocoons were irregular in shape and had thin filaments entwined on the outside. The duration of the pupal stage was approximately 1.87 ± 0.35 days in the 27 °C treatment.

For adult stage, *C. chilonis* adults emerged from cocoons by drilling out of one end (Fig. 1J), and eclosion generally happened during the daylight hours. The pupal stage of males was slightly shorter than females, and male adults generally emerged first. *C. chilonis* males and females can mate within 24 h after eclosion (Fig. 1L); and both sexes can mate multiple times. Repeated experiments showed that parthenogenesis is possible, and unfertilized eggs develop solely into males. The duration of the adult stage was 5.28 ± 0.07 days at 27 °C. The number of female *C. chilonis* produced by a single parasitized *C. suppressalis* was 24.57 ± 4.64 at 27 °C, whereas the number of males was 9.00 ± 1.59 at 27 °C (Table 3). The ratio of female to male adults was 2.85 ± 0.29 and the emergence rate was 0.82 ± 0.06% at 27 °C (Table 3).

**Table 3:** Developmental indicators of *Cotesia chilonis* adults.

	27 °C	36 °C
Number of cocoons	42.00 ± 6.87	23.14 ± 1.92
Number of females	24.57 ± 4.64	12.50 ± 4.50
Number of males	9.00 ± 1.59	3.50 ± 0.50
Number of adults	33.57 ± 6.04	16.00 ± 4.00
Ratio of female to male	2.85 ± 0.29	3.83 ± 1.83
Emergence rate (%)	0.82 ± 0.06	0.57 ± 0.12

### The effect on the developmental index of *C. chilonis* under high temperature

Different developmental stages of *C. chilonis* were all observed and recorded by microscopy after parasitism at 27 and 36 °C. The developmental duration of eggs showed little difference between 27 and 36 °C (*t* = −5.511, *P* < 0.001). However, the duration of the 1^st^ and 2^nd^ larval instars were longer in the 36 °C treatment as compared to 27 °C (1^st^ instar larvae: *t* = −3.177, *P* < 0.05; 2^nd^ instar larvae: *t* = −1.949, *P* < 0.05), whereas the 3^rd^ larval instar, pupal and adult stages were shorter at 36 °C (3^rd^ instar larvae: *t* = 4,627, *P* < 0.001; pupal stage: *t* = 3.984, *p* = 0.003; adult stage: *t* = 7.162, *P* < 0.001) (Table 2). Moreover, the treatment of 36 °C resulted in the death of samples, in the remaining two survival treatments, the number of cocoons, female and male *C. chilonis* produced by a single parasitic *C. suppressalis* was obviously less at 36 °C, and the number of adults produced at 27 °C was about twice the number at 36 °C, indicating that high temperature stress leads to a decrease in cocoon numbers and offspring (Table 3). Furthermore, the results showed that exposure to 36 °C caused a decrease in the emergence rate and an increase in the ratio of female to male adults.

**Figure 2:**
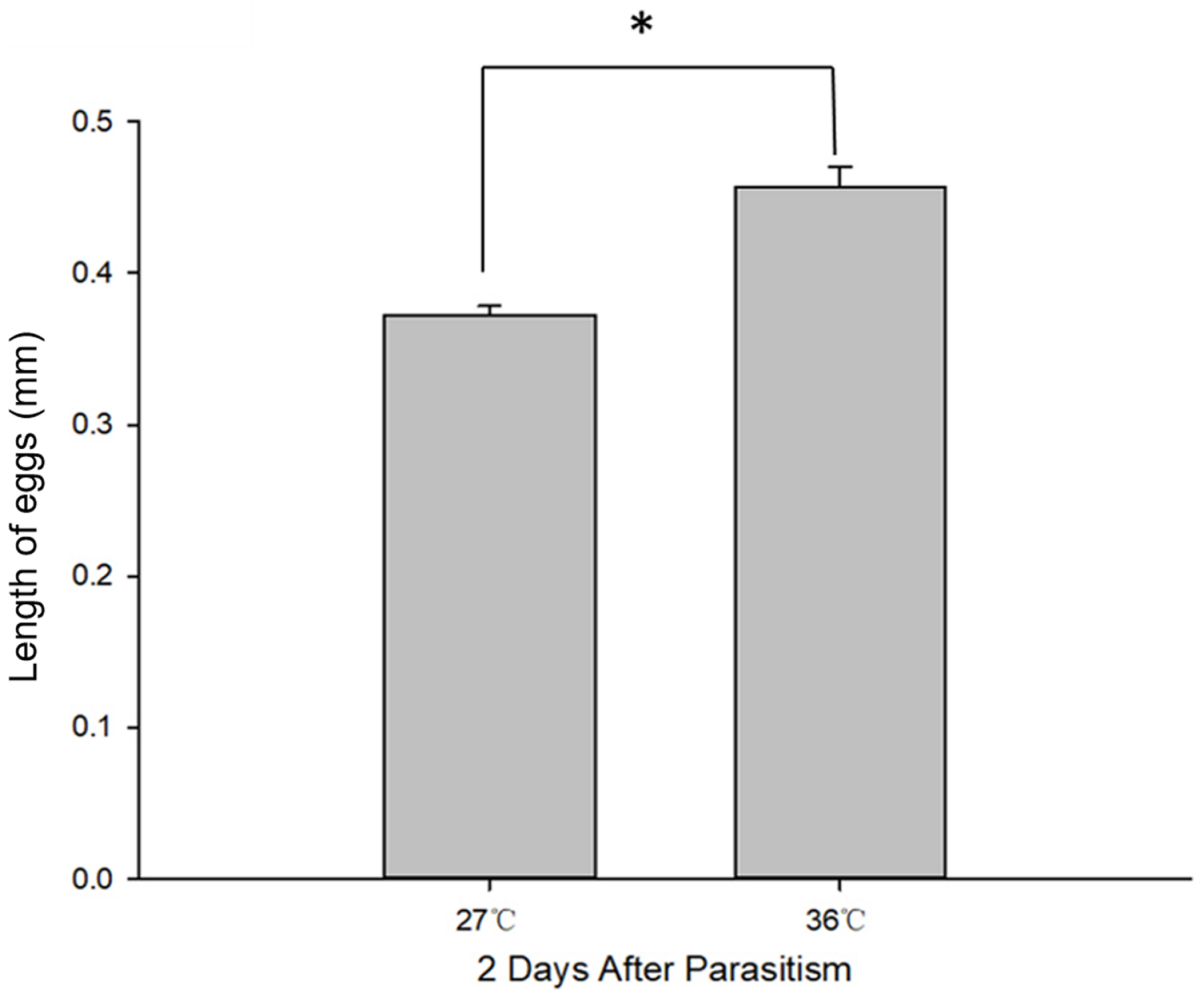
*C. chilonis* body length (mm) in the 27 and 36 °C treatments. Statistics represent means ± SE at two days of parasitism. Data were analyzed using independent samples *t*-test built in SPSS software. *P* < 0.05 was considered statistically significant. Asterisks represent significant differences between 27 and 36 °C.

**Figure 3:**
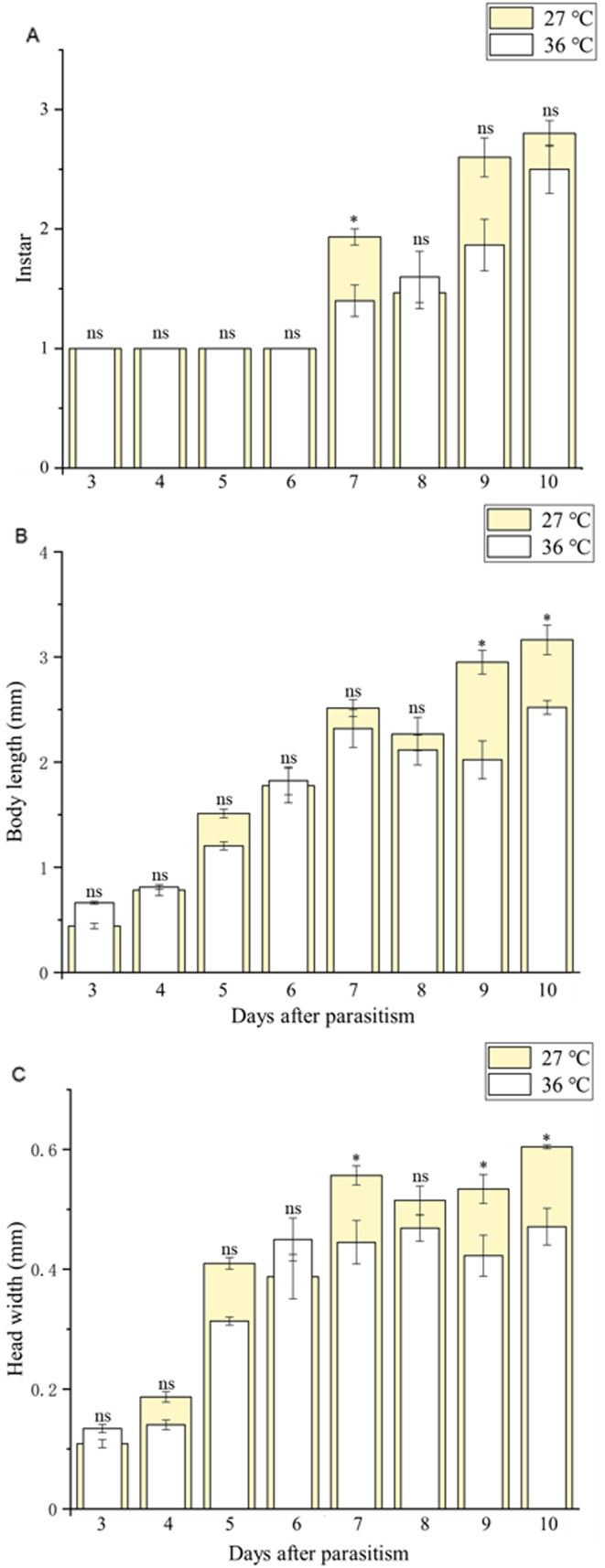
Larval instar stages and growth measurements of *C. chilonis* during parasitism. (A) Instar stage, (B) larval body length (mm) and (C) larval head width (mm) in insects maintained at 27 °C or 36 °C. Measurements were taken at 24-h intervals from 3–10 days after parasitism. All statistics are presented as means ± SE, and data were analyzed by the independent samples *t*-test, *P* < 0.05. Asterisks represent significant differences between 27 and 36 °C; ns indicates no significant differences.

Body length of 1^st^, 2^nd^ and 3^rd^ larvae instars were almost identical at 36 and 27 °C (1^st^ instar larvae: *t* = 0.064, *P* = 0.949; 2^nd^ instar larvae: *t* = 0.137, *P* = 0.892; 3^rd^ instar larvae: *t* = 1.405, *P* = 0.173) and so was head width (1^st^ instar larvae: *t* = 0.510, *P* = 0.611; 2^nd^ instar larvae: *t* = 0.728, *P* = 0.470; 3^rd^ instar larvae: *t* = 1.140, *P* = 0.265) (Table 2), while the length of egg (Fig. 2) was significantly longer in the 36 °C treatment as compared to 27 °C (*t* = −5.926, *P* < 0.001). Interestingly, we observed different developmental stages of *C. chilonis* larvae within the same *C. suppressalis* on the same day of dissection except that *C. chilonis* was in the 1^st^ larval instar stage on the 3^rd^, 4^th^, 5^th^ and 6^th^ days after parasitism regardless of temperature (Fig. 3A). The body length of *C. chilonis* larvae in the 36 °C treatment was significantly shorter than the 27 °C treatment on the 9^th^ and 10^th^ days after parasitism (9^th^ day: *t* = 4.376, *P* < 0.001; 10^th^ day: *t* = 4.117, *P* = 0.002). Similarly, the head width of *C. chilonis* larvae was significantly shorter in the 36 °C treatment as compared to that in the 27 °C treatment on the 7^th^, 9^th^ and 10^th^ days after parasitism (7^th^ day: *t* = 2.806, *P* = 0.009; 9^th^ day: *t* = 2.649, *P* = 0.011; 10^th^ day: *t* = 4.347, *P* = 0.022). Therefore, the body length and head width of measured different instar *C. chilonis* larvae were generally shorter in insects receiving the 36 °C treatment as compared to that in the 27 °C treatment (Figs. 3B, 3C).

### Rate of larval instar development under normal and high temperature

**Figure 4:**
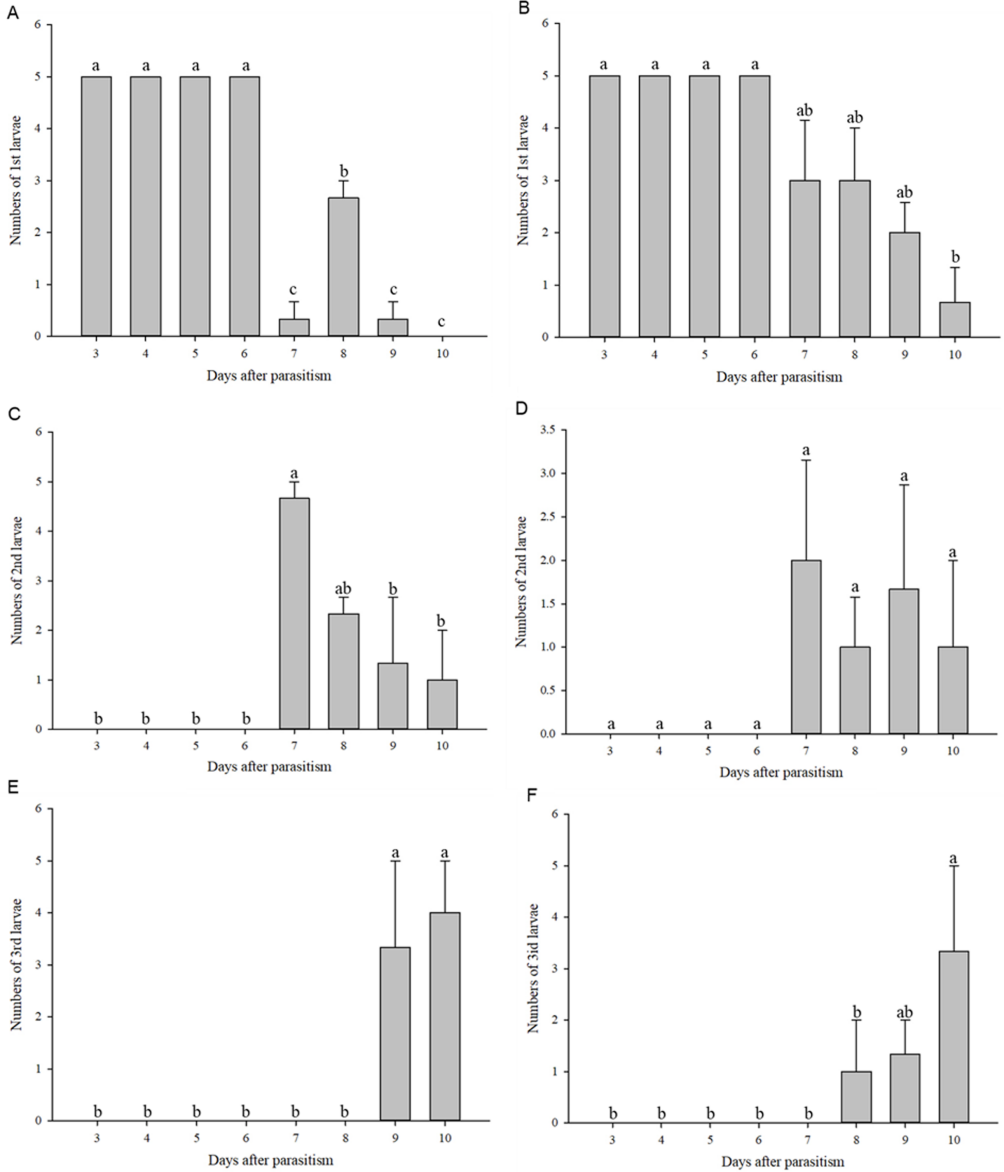
Numbers of larvae according to instar stage. Numbers of 1^st^ instar larvae at 27 °C (A); Numbers of 1^st^ instar larvae at 36 °C (B); Numbers of 2^nd^ instar larvae at 27 °C (C); Numbers of 2^nd^ instar larvae at 36 °C (D); Numbers of 3^rd^ instar larvae at 27 °C (E); Numbers of 3^rd^ instar larvae at 36 °C (F). Measurements were taken at 24-h intervals at days 3–10 after parasitism. Statistics represent means ± SE, and columns labeled with different letters indicate significant differences at 27 °C or 36 °C using one-way ANOVA followed by Tukey’s multiple comparison analysis (*P* < 0.05).

First instar larvae of *C. chilonis* appeared on the third day after parasitism in both the 27 and 36 °C treatments (Figs. 4A–4B). The number of 1^st^ instar larvae was lower in the 27 °C treatment as compared to 36 °C beginning at day seven after parasitism (27 °C: *F*_7,16_ = 134.667, *P* < 0.001; 36 °C: *F*_7,16_ = 7.245, *P* = 0.001).

Second instar larvae of *C. chilonis* began to appear on the seventh day after parasitism (Figs. 4C–4D). Regardless of temperature, numbers of 2^nd^ instar larvae were high on the 7^th^ day after parasitism and began to decline on the 8^th^ day after parasitism (Figs. 4C–4D). Third instar larvae of *C. chilonis* appeared on the eighth and ninth days of parasitism for treatments at 36 and 27 °C, respectively, and remained elevated through day 10 (Figs. 4E–4F).

### Expression of *Cchsp11.0* and *Cchsf* at different developmental stages after high temperature treatment

**Figure 5:**
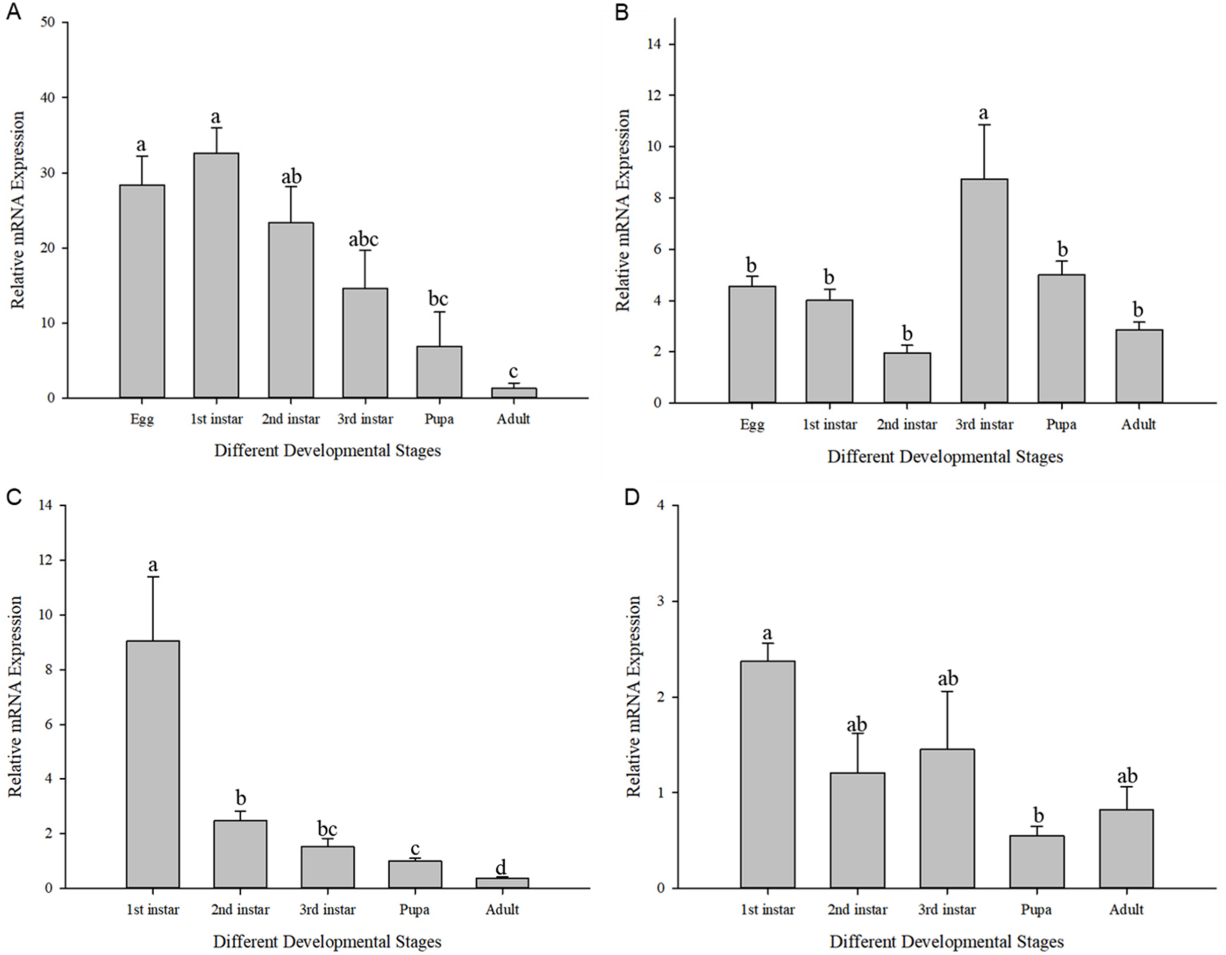
Relative mRNA expression levels at different developmental stages. (A) *Cchsp11.0* at 27 °C; (B) *Cchsf* at 27 °C; (C) *Cchsp11.0* at 36 °C; (D) *Cchsf* at 36 °C. Statistics represent means±SE, and columns labeled with different letters indicate significance between developmental stages using one-way ANOVA followed by Tukey’s multiple comparison analysis (*P* < 0.05).

The expression of *Cchsp11.0* and *Cchsf* showed divergent expression patterns in different developmental stages at 27 and 36 °C (27 °C: *Cchsp11.0*: *F*_11,16_ = 8.495, *P* = 0.002; *Cchsf*: *F*_11,16_ = 14.111, *P* = 0.001; 36 °C: *Cchsp11.0*: *F*_13,17_ = 48.627, *P* < 0.001; *Cchsf*: *F*_11,15_ = 3.419, *P* = 0.048). At 27 °C, *Cchsp11.0* expression was highest in the 1^st^ larvae instar stage and lowest in the adult stage (Fig. 5A), while *Cchsf* was highest in the 3^rd^ larvae instar stage and lowest in the 2^nd^ larvae instar stage (Fig. 5B). At 36 °C, the expression of *Cchsp11.0* and *Cchsf* was highest in the 1^st^ larvae instar stage and lowest in the adult and pupal stages, respectively (Figs. 5C–5D). Regardless of temperature, *Cchsp11.0* expression showed a decreasing trend after the 1^st^ instar stage.

**Figure 6:**
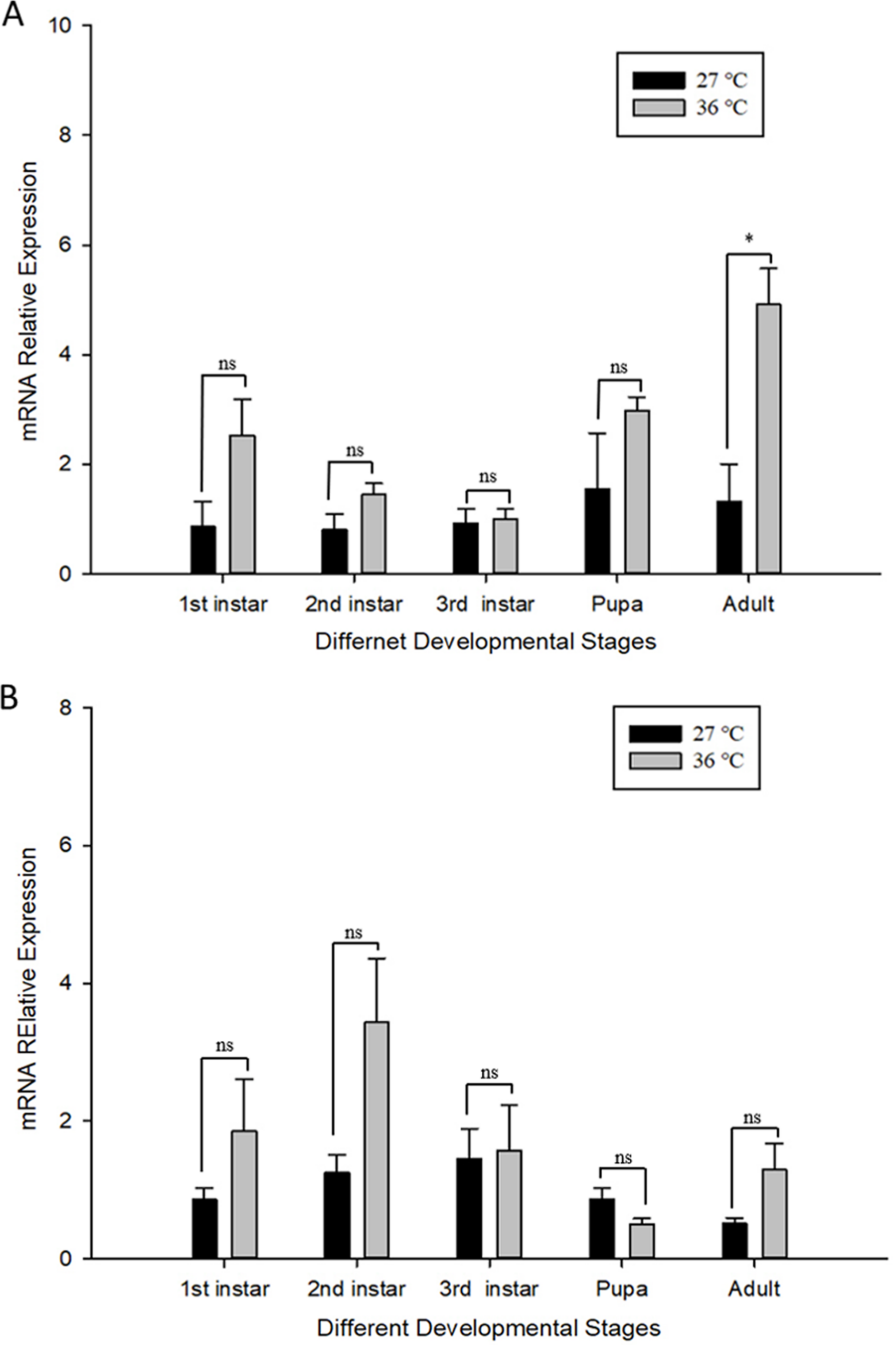
Relative mRNA expression levels of *Ccshsp11.0* (A) and *Cchsf* (B) at different developmental stages at 27 and 36 °C. Statistics represent means ± SE, and data were analyzed by the independent samples t-test, *P* < 0.05. Asterisks represent significant differences between 27 and 36 °C; ns indicates no significant differences.

The influence of high temperature on the expression of *Cchsp11.0* and *Cchsf* is not consistent (Figs. 6A–6B). The relative expression of *Cchsp11.0* was significantly up-regulated at the adult stage at 36 °C and was 3.71-fold higher than expression at 27 °C (*t* = −3.745, *P* = 0.013), whereas *Cchsf* was not remarkably sensitive to 36 °C.

## Discussion

*C. chilonis* is the primary endoparasitic wasp of larval stages of *C. suppressalis* and a potential biocontrol species (Huang, Wu & Ye, 2011; Wu et al., 2013). Due to the low immunity, high mortality and the low cocooning percentage, 3^rd^ instar larvae of *C. chilonis* were chosen and used in this study (Hang & Lin, 1989; Li, 2011). Giant cells started to appear on the second of parasitism by syngamy and on the third day by parthenogenesis, which is consistent with previous research (Hang, 1991). We observed different larval instars of *C. chilonis* were present in a single *C. suppressalis*; this likely due competition between wasps, resulting in a 1–2 day gap in development (Hang, Shen & Lu, 1993).

Temperature has a huge influence on the regulation of physiological functions in insects, including growth and reproduction (Adamo et al., 2012; Huey et al., 2012). Our results showed that the developmental duration of *C. chilonis* was generally shortened in response to high temperatures, which is consistent with results for parasitic wasps in the Braconidae (Liang et al., 2007; Gao, Huang & Chen, 2003; Du, Shen & Wang, 2009). For example, generational development in *Diachasmimorpha longicaudata* was shortened with increasing temperature from 45.7 d at 15 °C to 15.2 d at 30 °C (Liu, Chen & Zeng, 2012). In our study, the developmental duration of the egg stage showed little difference between 27 and 36 °C, and that of the 1^st^ and 2^nd^ instar larvae was slightly prolonged under high temperature treatment (see Table 2), which may be the reason that exposure of eggs to extreme temperatures can negatively affect larval growth and phenotypic plasticity (Potter, Davidowitz & Arthur Woods, 2011). The body length and head width of *C. chilonis* larvae were reduced at 36 °C but the difference was not significant (see Table 2), which was also true for *Psyttalia incise* (Liang et al., 2007). Body length and head width of different larval instars were significantly different at 27 and 36 °C on the 9^th^ and 10^th^ days of parasitism, and numbers of 3^rd^ instar larvae appeared earlier at 36 °C vs. 27 °C. In general, our results indicate that high temperature inhibited growth and development. With respect to reproduction, cocoon numbers were slightly reduced at 36 °C, while the emergence rate of *C. chilonis* was significantly lower (see Table 3). Although the number of males, females and adults declined in the 36 °C treatment relative to 27 °C, the ratio of females to males increased at 36 °C, which agrees with results obtained for *Spathius agrili* (Tian, Wang & Yang, 2009).

The influence of temperature on insect growth and reproduction also impacts behavior, longevity, and survival (Kingsolver, Higgins & Augustine, 2015; Roitberg & Mangel, 2016) and includes the higher mortality and shorter adult lifespan under high temperature treatment (Chen et al., 2018; Zheng et al., 2017). In response to high temperature, insects react with changes in behavior, metabolism, and development. For example, high temperatures increase metabolism and oxygen demands, resulting in the production of more free radicals and the formation of toxic products which trigger a defensive response (Colinet et al., 2015). Moreover, when external temperatures increase, insects produce HSPs to prevent denaturation of proteins that do not function well at high temperatures (González-Tokman et al., 2020), and ; this may explain why *Cchsp11.0* expression was significantly up-regulated at the adult stage at 36 °C. Furthermore, *Cchsp11.0* and *Cchsf* expression was highest in the 1^st^ larvae instar stage at 36 °C, indicating that the reaction to high temperatures was strongest in the L1 stage. *Cchsp11.0* expression was gradually down-regulated at 27 and 36 °C as *C. chilonis* matured, possibly because of the adaptation to temperature. However, expression patterns for *Cchsf* did not correlate with *Cchsp11.0*, which warrants further study.

## Conclusions

The duration of development, morphology, emergence rate, numbers of offspring and ratio of females to males are significant indicators for quality control during artificial breeding of *C. chilonis*. High temperatures increased the ratio of females to males and generally inhibited the growth and reproduction of *C. chilonis*. Insect developmental stages differ in vulnerability to high temperature (Bowler & Terblanche, 2008). *Cchsp11.0* and *Cchsf* play different roles in different developmental stages of *C. chilonis* at normal and high temperature. With the encroachment of global warming, we call on everyone to pay more attention to the impact of extreme weather on the growth and reproduction of insect natural enemies and to find pragmatic solutions to protect them.

## Supplemental Information

10.7717/peerj.11353/supp-1 Supplemental Information 1 Raw measurements.

10.7717/peerj.11353/supp-2 Supplemental Information 2 qPCR results.

## Additional Information and Declarations

### Data Availability

The following information was supplied regarding data availability: The raw measurements are provided in the Supplementary Files.
